# Targeted Gut Microbiome Intervention to Reduce Anastomotic Leak in Colorectal Cancer Surgery: A Narrative Review and Potential Recommendations

**DOI:** 10.3390/cancers18132181

**Published:** 2026-07-07

**Authors:** Ana Grigoraș, Bogdan Filip, Mihaela-Mădalina Gavrilescu, Dragos-Viorel Scripcariu, Ionuț Huțanu, Maria-Gabriela Aniței, Viorel Scripcariu

**Affiliations:** 1 Surgical Department, Grigore T. Popa University of Medicine and Pharmacy, 700115 Iași, Romania; grigoras.ana@umfiasi.ro (A.G.); gavrilescu.mihaela@umfiasi.ro (M.-M.G.); dscripcariu@gmail.com (D.-V.S.); ionut.hutanu@umfiasi.ro (I.H.); maria-gabriela.anitei@umfiasi.ro (M.-G.A.); viorel.scripcariu@umfiasi.ro (V.S.); 2 I-st Oncological Surgical Unit, Regional Institute of Oncology, 700483 Iași, Romania

**Keywords:** colorectal cancer, anastomotic leakage, probiotics, gut microbiome, colorectal surgery, synbiotics, perioperative care, postoperative complications

## Abstract

**Background/Objectives**: Anastomotic leakage (AL) remains one of the most severe postoperative complications following colorectal surgery and is associated with increased morbidity, delayed adjuvant therapy, and impaired oncological outcomes in CRC patients. Increasing evidence suggests that alterations in the gut microbiome contribute to the pathogenesis of AL through effects on epithelial integrity, collagen metabolism, inflammatory pathways, and immune regulation. This review aimed to provide an updated overview of AL in CRC patients and to evaluate current evidence regarding the perioperative use of probiotics, prebiotics, and synbiotics as microbiome-modulating interventions in reducing AL after radical CRC surgery. **Methods**: A comprehensive literature review of randomized controlled trials (RCTs) investigating perioperative microbiome-targeted interventions in CRC patients undergoing colorectal surgery was conducted. Searches of PubMed, Embase, Cochrane Library, Scopus, and Clarivate databases identified 477 records, of which 21 RCTs met the inclusion criteria and were included in the final analysis. **Results**: Current evidence supports the role of probiotics in modulating postoperative immune and inflammatory responses. Four studies demonstrated a statistically significant reduction in AL incidence. Perioperative probiotic administration was additionally associated with lower rates of infectious complications, attenuation of systemic inflammatory responses, and earlier recovery of bowel function. Multi-strain formulations containing *Lactobacillus* and *Bifidobacterium* species appeared particularly promising. Overall, microbiome-targeted interventions were safe and well tolerated. However, treatment efficacy varied according to bacterial strain composition, dosage, and timing of administration. **Conclusions**: Perioperative microbiome modulation may contribute to restoration of gut microbial diversity and reduction in AL risk after radical CRC surgery. Multi-strain formulations containing *Lactobacillus* and *Bifidobacterium* species appear particularly promising. Nevertheless, further large-scale, standardized clinical trials are required before definitive recommendations can be established.

## Simple Summary

In the particular context of colorectal cancer (CRC), a serious postoperative complication such as anastomotic leak, may disrupt the planned multimodal treatment strategy and adversely affect oncological outcomes and increase the risk of cancer recurrence. Evidence from the literature provides substantial support for the role of probiotics in modulating postoperative immune and inflammatory responses. In order to establish a valid recommendation for a targeted microbiota intervention as a preventive measure, an extensive search of the current literature is needed, and mechanical bowel preparation, oral antibiotics decontamination, prebiotics and probiotics may be considered as eligible interventions. International practical guidelines for the clinical use of probiotics and prebiotics restrict their recommendation to postoperative septicemia and infectious complications with a level of evidence of 3. This review provides a framework for the development of future clinical studies evaluating standardized targeted microbiota interventions aimed at preventing postoperative complications, with particular emphasis on anastomotic leakage in patients undergoing CRC surgery.

## 1. Introduction

Colorectal cancer (CRC) remains a major focus in surgical practice, with significant advances achieved in recent years owing to its high incidence and the continuous refinement of surgical techniques, perioperative management, and preventive strategies for postoperative complications. Despite these developments, anastomotic leakage (AL) continues to represent one of the most severe postoperative complications following colorectal surgery. Reported incidence rates range from 1.5% to 23%, and it is associated with substantial morbidity and mortality, with mortality rates reaching 16–29% [1], regardless of the underlying independent or associated risk factors. Additionally, surgical site infections remain the most common healthcare-associated infections worldwide [2].

In the particular context of CRC, many aspects need to be taken into consideration such as: a tumor-induced immunocompromised status; the treatment plan, which can include neoadjuvant therapy (radiotherapy/chemotherapy/immunotherapy); and the potential repercussions of postoperative complications on the oncological outcome—delaying adjuvant treatment, increased risk of recurrence, etc. [3].

A subclinical pro-inflammatory state within the colonic mucosa of patients with surgical treatment for CRC has been associated with impaired postoperative recovery of the intestinal barrier. This inflammatory microenvironment appears to be mediated by the gut microbiota, as transplantation of this patient-derived microbiota into murine models replicates the upregulation of multiple pro-inflammatory cytokines in the colonic tissues [4]. Furthermore, Hajar et al. demonstrated that specific bacterial strains can modulate cytokine expression in both the mucosal compartment and the intraluminal environment, occasionally contributing to heightened systemic inflammatory activity prior to surgery, as reflected by increased circulating leukocyte counts [3].

Increasing evidence suggests that the gut microbiome plays a causal and mechanistic role in CRC initiation, progression, and therapeutic response. At the time of diagnosis, patients with CRC already exhibit a distinct microbial signature, typically characterized by an enrichment of pathobionts such as enterotoxigenic Bacteroides fragilis, pks^+^ *Escherichia coli*, and *Fusobacterium nucleatum*, and, accompanied by a depletion of protective short-chain-fatty-acid-producing commensals. These alterations promote carcinogenesis through mechanisms including genotoxin-induced DNA damage, disruption of the epithelial barrier, metabolic reprogramming, and chronic inflammation, which together contribute to immune suppression and tumor progression [5].

The human body coexists in a symbiotic association with its microbiota, which functions as an integral biological ecosystem. In the surgical setting, the epithelial layer serves as a key defensive barrier against gut-derived pathogens, and its proper sealing is generally presumed to safeguard the underlying submucosal matrix from injurious inflammatory signals. However, this protective role may be altered in postoperative pathological conditions, potentially permitting sustained exposure of deeper tissues to inflammatory mediators [3,6]. Emerging evidence suggests that the gut microbiota may influence the risk of AL through effects on collagen preservation at the anastomotic site. The proposed mechanisms are multifaceted and include, in particular, the upregulation of matrix metalloproteinase-9 (MMP-9), a collagenolytic enzyme implicated in the degradation of anastomotic collagen and the development of AL. This enzymatic activity is accompanied by extracellular matrix breakdown at the anastomotic interface, thereby disrupting tissue integrity and impairing effective wound healing [3].

Recently, the use of probiotics as a complementary therapy in CRC has attracted increasing attention, due to their potential to modulate the gut microbiota and to enhance host immune responses. Engineered probiotics may provide advantages in CRC treatment by selectively targeting cancer cells and delivering therapeutic agents directly within the tumor microenvironment [7]. Several studies have evaluated probiotics, prebiotics, and synbiotics as perioperative strategies for the reduction or prevention of postoperative complications following colorectal surgery. The rationale for their use is supported by evidence derived from observational studies, case–control analyses, and RCTs, which collectively provide clinically relevant, real-world data. However, these studies are generally limited by small sample sizes and considerable heterogeneity, encompassing variations in study objectives, types of interventions, and reported outcome [8].

Therefore, the aim of this study is to evaluate current recommendations regarding the administration of probiotics, prebiotics and synbiotics as targeted interventions of the gut microbiome in the perioperative period of radical surgical treatment of CRC patients as a strategy for improving anastomotic healing and reducing the risk of anastomotic leakage. This narrative review explores the effects of microbiota-targeted interventions in order to identify the most promising strategies for a future prospective randomized clinical trial on CRC patients with the main objective of reducing postoperative complications and especially AL. It also analyzes the effects on gastrointestinal motility based on the most frequent adverse effects of these types of interventions, including alteration of intestinal transit.

## 2. An Overview of the Current Literature

1. AL in CRC
  - Definition
  - Characteristics
  - Manifestations
  - Diagnosis
  - Risk factors
2. Correlation between gut microbiota and anastomotic leak

**Mechanisms:**

- Microbiome-derived metabolites (SCFAs) and epithelial proliferation.
- Reinforcement of the submucosa.
- Maintenance of the collagen layer.
- Modulation of the inflammatory response.
- Alterations in microbiome composition (microbial shifts).

**Microbiota composition:**

- Bacteria in the gut microbiota of CRC patients who develop AL:
- Collagenase-producing: *Enterococcus faecalis*, *Clostridium difficile*, *Klebsiella pneumoniae*, *Pseudomonas aeruginosa*, *Proteus mirabilis*, and *Bacteroides* spp.
- *Alistipes onderdonkii*.
- Bacteria in the gut microbiota of CRC patients with favorable postoperative outcome:
- *Parabacteroides goldsteinii*.
- *Lactobacillus* spp., *Bifidobacterium*.
- Sources of dysbiosis.

3. Intervention in Gut Microbiota

**Mechanical bowel preparation**—may be associated with selective oral antibiotic therapy for bowel decontamination.

**Microbiota-targeted intervention—prebiotics/probiotics/synbiotics.**

- International Society for Probiotics and Prebiotics definition.
- Strains of protective bacteria and mechanisms.
- Formulation, type, and timing.
- **(WGO) Global Guidelines—Clinical Guides to Probiotic Products—official recommendations.**

### 2.1. Anastomotic Leakage in CRC

The international consensus on reporting anastomotic leaks after colorectal cancer surgery (CoReAL) established a definitive definition of AL as any extravasation or loss of integrity of the anastomosis, including dehiscence, insufficiency, failure, rupture, defect, or separation, regardless of the diagnostic modality used (radiologic, endoscopic, or intraoperative) and irrespective of clinical or biochemical manifestations [1].

The inconsistent reporting of critical information regarding AL compromises the validity of clinical trials and limits comparability across studies evaluating interventions targeting AL outcomes, thereby impeding the accurate assessment of strategies aimed at reducing its incidence and impact [9]. The expert panel of surgeons in the CoReAL group mitigated to standardize the reporting of AL following left-sided CRC resections, drawing on high-quality published evidence and patient perspectives [1].

The most widely adopted definition of AL was proposed by the International Study Group of Rectal Cancer (ISREC) [10], according to which it represents a defect in the integrity of the intestinal wall at the colorectal or coloanal anastomotic site, including suture or staple lines of neorectal reservoirs, resulting in a communication between the intra- and extraluminal compartments. A pelvic abscess adjacent to the anastomosis is also considered a manifestation of AL. Severity is classified into three grades: Grade A, characterized by the absence of overt clinical signs or symptoms and not requiring active therapeutic intervention; Grade B, involving clinical manifestations requiring active therapeutic management without relaparotomy; and Grade C, associated with abdominal sepsis and signs of peritoneal irritation necessitating relaparotomy [11,12].

The reported incidence of AL varies according to the anatomical location of the anastomosis. Ileocolic anastomoses are associated with a relatively low leak risk of approximately 1–4%, whereas colorectal anastomoses have reported leak rates ranging from 0.5% to 18%, and coloanal anastomoses from 5% to 19% [9]. The 2020 Consensus Committee on the Definition of Anastomotic Leak recommended that colonic and rectal AL should be considered distinct clinical entities due to differences in anatomy, surgical technique, and intestinal microbiota composition [13].

The diagnosis of AL relies on an integrated assessment of clinical manifestations, laboratory findings, imaging studies, and, when necessary, surgical exploration. AL should be suspected in the presence of recurrent fever, persistent abdominal pain, or signs of peritoneal irritation associated with elevated inflammatory markers; changes in postoperative drainage, particularly feculent or purulent discharge, or similar secretions at the surgical wound site; suggestive imaging findings; or direct identification of the leak through digital examination, colonoscopy, or surgical exploration [2]. Tachycardia and clinical deterioration should also prompt suspicion of AL imposing future investigations [13,14].

AL is most commonly diagnosed between postoperative days 5 and 8 [15]. Depending on the timing of diagnosis or the onset of AL-related complications, leaks may be classified as early or late onset, with late AL generally defined as occurring more than 30 days after surgery [15], or after 90 days according to the CoReAL consensus [1]. Nevertheless, clinical manifestations of AL are frequently absent before postoperative day 5 [10].

The most reliable biomarkers for the diagnosis of AL are C-reactive protein (CRP), procalcitonin (PCT), and leukocytosis when assessed dynamically and in combination, although CRP and leukocytosis alone are also considered acceptable indicators [16]. Radiological findings, preferably obtained by CT, include extraluminal leakage of contrast material administered by enema, localized perianastomotic collections, presacral abscess adjacent to the anastomosis, and the presence of perianastomotic or free intraperitoneal air [8,13].

In cases requiring surgical reintervention, AL may be identified by necrosis of the anastomotic edge, necrosis of a blind bowel loop, anastomotic dehiscence, and signs of peritonitis, in accordance with the consensus definition of colorectal AL [10,13].

The repercussions and complications associated with AL extend across multiple domains, including surgical outcomes, postoperative clinical evolution, and oncological prognosis [17]. Morbidity remains substantial, with the risk of permanent stoma formation exceeding 25% in some series [18]. Mortality among CRC patients who develop AL has been reported to range between 25% and 66% [19].

Risk factors for AL may be classified as modifiable (which can be influenced through patient-related measures or medical interventions) or non-modifiable, with several factors also identified as independent predictors of AL occurrence [10]. General risk factors associated with AL include male sex, considered an independent predictor; advanced age; poor overall patient status; associated comorbidities, smoking, obesity, and malnutrition; and laboratory abnormalities such as leukocytosis, anemia, and hypoalbuminemia. Neoadjuvant therapies, including chemotherapy and radiotherapy, have also been identified as independent risk factors. Local factors include disease-specific characteristics such as tumor stage and location, as well as local complications including peritumoral abscess, bowel obstruction, intestinal perforation, and peritoneal sepsis. Surgery-related factors encompass operative duration, surgical technique, adequacy of local vascularization at the bowel ends, tension at the anastomotic site, and intraoperative blood loss or transfusion requirements [2,20].

Palmisano and colleagues reported that the intestinal microbiota of patients with colorectal cancer differs significantly from that of healthy individuals. Moreover, they suggested that the presence of aggressive bacterial strains in the preoperative period, together with the depletion of protective commensal bacteria, supports the hypothesis that a distinct microbiota composition may constitute a risk factor for the development of anastomotic leakage [21].

As AL may still occur in patients who do not present the established risk factors, increasing attention has been directed toward the relationship between the intestinal microbiota and the development of AL, with particular emphasis on intestinal dysbiosis and on the strain-specific bacterial mechanisms capable of disrupting the healing process of intestinal anastomoses.

### 2.2. The Correlation Between Gut Microbiota and Anastomotic Leak

The correlation involves the impact of microbiome-derived metabolites on epithelial proliferation, maintenance of the collagen layer, and reinforcement of the submucosa, as well as modulation of the inflammatory response and alterations in microbiome composition (microbial shifts).

**Mechanisms.** According to Bartolini et al., four key conditions are required for the development of AL: the presence of collagenase-producing bacteria, intestinal dysbiosis, a detectable inflammatory response at the anastomotic site triggered by pathogens, and sufficient bacterial virulence to overcome host defense mechanisms [6,22]. In the surgical context, although the epithelial layer serves as a crucial barrier against gut pathogens and its integrity, it is expected to protect the underlying submucosal matrix from harmful inflammatory stimuli; this protective function may be insufficient, allowing continued exposure of deeper tissues to inflammatory mediators [3,6].

Epithelial regeneration relies on the availability of nutritional substrates, particularly those derived from the gut microbiota. Among these, short-chain fatty acids (SCFAs) are key microbial metabolites that contribute to epithelial integrity and strengthen gut barrier function. SCFAs are produced by colonic bacteria through the fermentation of dietary fibers that are not digested in the small intestine and are mainly metabolized in the cecum and proximal colon. This process yields the primary SCFAs—acetate, propionate, and butyrate—which constitute the dominant forms present within the large intestine. A strategy to shift the colonic microbiota toward a butyrogenic profile with increased SCFA production involves dietary supplementation with fermentable fibers, particularly oligosaccharides such as inulin and galacto-oligosaccharides (GOS) [6]. In the experimental model developed by Hajar et al. where they supplemented the diet for 2 weeks before surgical moment with inulin or GOS, they found increased levels of SCFAs in the gut and improved macroscopic healing of the anastomosis [4]. The findings were confirmed by a pathologist who was blinded to the intervention group, who observed enhanced re-epithelialization and improved mucosal continuity in mice receiving oligosaccharide supplementation. In addition, immunohistochemical analysis demonstrated increased expression of the Ki-67 proliferation marker at the wound site in mice supplemented with inulin, indicating heightened cellular proliferation [6].

Bacterial metabolites, particularly SCFAs, may also contribute to the preservation of the collagen matrix and reinforcement of the submucosa following surgical injury. In a different experimental model, mice receiving diets supplemented with inulin and GOS exhibited increased collagen content at the anastomotic site, objectivated through higher hydroxyproline levels, and reduced collagenolytic activity within the wound area [4]. Butyrate plays a special role as a modulator of local and systemic immunity in the specific context of CRC as it stimulates proliferation in normal colonocytes while inhibiting the growth of malignant cells, a phenomenon commonly referred to as the “butyrate paradox”. Under physiological conditions, colonocytes utilize butyrate through mitochondrial oxidation. In CRC cells, a metabolic shift toward aerobic glycolysis reduces butyrate utilization, leading to its intracellular accumulation. Butyrate then acts as a histone deacetylase (HDAC) inhibitor, modulating gene expression by suppressing proto-oncogene activity and promoting tumor suppressor gene expression [23,24]. Prominent butyrate-producing species are *Eubacterium hallii*, *Faecalibacterium prausnitzii*, *Roseburia intestinalis*, and *Eubacterium rectale* [4].

The submucosa is considered to provide tensile strength and structural integrity to intestinal anastomosis, owing to its rich content of connective tissue and collagen, and is therefore regarded as a fundamental determinant of anastomotic healing [3].

Anastomotic healing is a complex process that can be divided into four partially overlapping phases: hemostasis, inflammation, proliferation (including cellular infiltration, angiogenesis, collagen matrix formation and re-epithelialization), and maturation/remodeling. Collagen plays a central role in regulating these stages, and disturbances in collagen synthesis or degradation may impair wound healing and promote persistent inflammation. Delayed healing is associated with increased levels of matrix metalloproteinases and other collagenolytic enzymes within the extracellular matrix, leading to degradation of its structural components and dysregulated activation of soluble mediators involved in tissue repair [22].

The extracellular matrix is affected by both endogenous enzymes released from perianastomotic intestinal cells and exogenous enzymes produced by bacteria belonging to the gut microbiota. Intestinal wall repair and microbiota adaptation are normally maintained in a state of dynamic homeostasis, while reduced microbial diversity has been inversely associated with the occurrence of AL [3]. Reduced microbial diversity favors the overgrowth of pathogenic bacteria and depletion of beneficial microbial populations, resulting in intestinal dysbiosis—a quantitative and qualitative imbalance of the gut microbiota associated with impaired intestinal barrier function and an increased risk of complications [2,25]. Van Praagh and colleagues observed that microbial diversity at the anastomotic site was diminished in patients who developed AL, promoting the proliferation of *Lachnospiraceae* spp. and *Bacteroidaceae* spp., bacterial taxa involved in mucin degradation. As mucin represents a major protective component of the intestinal mucus layer, its degradation may contribute to compromised mucosal integrity and impaired anastomotic healing [26].

Colorectal segment resection results in exposure of the intestinal lumen to oxygen, while vascular ligation causes a transient interruption of local blood supply. This shift modifies the oxygen tension within the normally anaerobic intestinal environment, thereby inducing alterations in the balance between obligate and facultative anaerobic bacterial populations. Furthermore, ischemia and subsequent reperfusion at the local level are associated with a decrease in the relative abundance of *Lactobacillus* species and an increase in *Escherichia coli* [27].

**Microbiota composition.** The proposed hypothesis suggests that the healing process at the anastomotic site promotes selective local proliferation of bacterial species such as *Enterococcus* spp. and other collagenase- and protease-producing organisms classically associated with AL, potentially driven by collagen deposition from human fibroblasts. This is supported by the absence of *Enterococcus* spp. in mucosal biopsy samples obtained at the time of surgery, followed by its detection after the diagnosis of AL [28]. A summary is given in Table 1.

The most relevant collagenase-producing bacteria identified in the gut microbiota of CRC patients who develop AL include *Enterococcus faecalis*, *Clostridium difficile*, *Klebsiella pneumoniae*, *Pseudomonas aeruginosa*, *Proteus mirabilis*, and *Bacteroides* spp. Jørgensen et al. conducted a systematic review evaluating the association between collagenase-producing bacteria and AL in colorectal surgery, reporting a higher frequency of such organisms in patients with AL. Collagenase production was documented in 10 of 15 included studies. In one study, collagenase-producing bacteria were identified in 14 of 19 cultures (73.7%), although specific strains were not specified. *Enterococcus faecalis* was the most frequently reported organism, followed by *Pseudomonas* spp., *Klebsiella* spp., and *Proteus* spp., with a predominance of *Pseudomonas aeruginosa* and *Klebsiella pneumoniae* in samples from patients with AL. Additionally, two studies identified *Clostridium difficile* infection as a primary outcome [20]. *Pseudomonas aeruginosa* and *Enterococcus faecalis* showed a marked collagenolytic activity, both being capable of degrading type I collagen. Collectively, these mechanisms promote excessive collagen breakdown and contribute to the development of AL [23].

Analysis of drain fluid samples has shown that nearly all patients with AL present positive cultures by postoperative day 5 [29], whereas most non-AL patients have negative cultures [30,31]. In contrast, Sparreboom et al. reported no significant differences in culture positivity between AL and non-AL patients on postoperative days 1 to 3 [32]. Similarly, Komen et al. observed that the absence of *Enterococcus faecalis* in drain fluid on postoperative day 3 has a high negative predictive value for AL [33]. Another study found an approximately fourfold increased risk of AL in patients with elevated levels of *Bifidobacterium* spp. in mucosal tissue adjacent to the anastomosis, potentially through impaired tissue perfusion and reduced oxygenation at the anastomotic site [33].

*Enterococcus faecalis* is recognized as a commensal member of the intestinal microbiota that can rapidly expand in the colon following antibiotic exposure. It may exert deleterious effects on anastomotic healing by selectively colonizing injured intestinal tissue and adopting a more virulent, collagenolytic phenotype. Within host tissue, it activates MMP-9 and binds and hyperactivates human plasminogen (PLG). The ability of the colonic immune system and commensal microbiota to eliminate resistant *E. faecalis* is further compromised following extensive depletion of the intestinal microbiota with broad-spectrum antibiotics [34,35].

*Pseudomonas aeruginosa* has been implicated as a risk factor for AL in the experimental study by Olivas et al., conducted on colotomized laboratory animals receiving either preoperative radiotherapy alone or radiotherapy combined with *P. aeruginosa* colonization. In this setting, a shift toward a more virulent bacterial phenotype was observed, characterized by increased collagenolytic activity, enhanced invasiveness, and epithelial cell damage [36]. Steyer et al. showed that the selective eradication of bacteria such as *Pseudomonas aeruginosa* and *Enterococcus faecalis* through locally acting antibiotic therapy provides indirect evidence that these pathogenic microorganisms play a significant role in the development of AL. They showed successful clinical avoidance of left-sided colorectal AL through transanal administration of targeted antibiotic mixture for local decontamination [37].

Similarly, Hajjar R. [4] published a 2023 murine study demonstrating that preoperative human-derived bacterial communities influence colonic anastomotic healing and AL development. Mice with depleted intestinal microbiota exhibited improved macroscopic anastomotic healing and increased extracellular matrix components at the anastomotic site, particularly hydroxyproline and fibronectin, reflecting enhanced collagen content. Moreover, the AL-associated patient phenotype—characterized by elevated preoperative mucosal pro-inflammatory cytokines—was transferable to mice via fecal microbiota transplantation (FMT), indicating that mucosal inflammatory regulation is microbiota-dependent. Within this model, *Alistipes onderdonkii* was associated with increased AL incidence, whereas *Parabacteroides goldsteinii* correlated with reduced AL risk. These bacterial species were shown to adhere to the colonic mucosa, resist both prophylactic antibiotics and mechanical bowel preparation (MBP), and directly influence postoperative surgical outcomes in murine models [4].

Certain bacterial species within the gut microbiota have been shown to exert beneficial effects on anastomotic healing. *Lactobacillus* spp. may enhance epithelial repair by stimulating the production of reactive oxygen species via NADPH oxidase 1 (NOX1), thereby promoting epithelial cell proliferation and migration and contributing to the maintenance of intestinal barrier integrity. In addition, *Lactobacillus* species can directly inhibit pathogenic bacteria [2].

The perioperative course of an oncologic patient submitted to colorectal surgery—from preoperative preparation to postoperative recovery—has a cumulative impact on the gut microbiota. Baseline composition is shaped by diet, lifestyle, and comorbidities. Neoadjuvant therapies and preoperative measures such as fasting, mechanical bowel preparation, oral antibiotics, and prophylactic intravenous antibiotics disrupt microbial balance, reducing luminal bacteria while altering mucosa-associated communities. Surgical intervention further modifies gastrointestinal physiology and microbiota composition, while postoperative complications—including infection, anastomotic leak, and dysmotility—are closely influenced by these perioperative microbial changes [27].

Postoperative physiological changes in the gastrointestinal tract are particularly pronounced in patients who develop postoperative complications and may persist for up to 24 months after surgery [38], and create conditions favorable for the overgrowth of potentially pathogenic organisms, including *Pseudomonas aeruginosa* and *Enterococcus faecalis*, or promote dominance of healthcare-associated pathogens such as *Clostridium difficile*. Consequently, the postoperative restoration of a balanced gut microbiota is considered an important aspect of recovery [27]. Restoration of a balanced gut microbiota may therefore support improved recovery prognosis in CRC patients.

### 2.3. Intervention in Gut Microbiota

The first evidence of bacterial involvement in the pathogenesis of anastomotic leakage was published in 1955, which led to the promotion of prophylactic oral antibiotic therapy as part of preparation for gastrointestinal surgery [2]. The efficacy of antibacterial treatment in reducing postoperative complication has been demonstrated in several studies, being considered from preoperative bowel preparation and perioperative infectious complications prevention, to treatment of postoperative complications. It is administered orally, intravenously or as local decontamination as a way of preventing anastomotic leak or surgical site infections.

**Mechanical bowel preparation.** For several years, the generally accepted bowel preparation in colorectal surgery consisted of routine preoperative MBP combined with oral antibiotics. Perioperative bowel preparation comprises three key elements: MBP, preoperative oral antibiotic (OAB) administration, and perioperative intravenous antibiotic therapy. Polyethylene glycol (PEG) and sodium phosphate are the most commonly utilized agents for mechanical bowel preparation.

Given the critical role of bacterial load and colonization at the time of surgery, numerous studies have highlighted the benefit of combining the two strategies (MBP + OAB) in reducing postoperative infectious complications, particularly AL. A 2023 pooled-analysis with trial sequential analysis developed by Yue et al., which explored the effect of MBP combined with oral antibiotic bowel decontamination versus MBP alone on patients undergoing bowel resection, demonstrated the importance of the association. They analyzed 22 RCTs, and prospective studies comprising 8852 patients were allocated into two groups according to the use of oral antibiotic decontamination in addition to mechanical bowel preparation and demonstrated a significantly lower AL rate when associating the two: OR = 0.43, 95% CI: 0.23–0.81, *p* = 0.009, I^2^ = 73% [39].

Furthermore, when comparing the association of MBP and OAB decontamination to only MBP and to no bowel preparation, an international, multi-center, prospective audit published in 2018 coordinated by the 2017 European Society of Coloproctology (ESCP) collaborating group found that the association of MBP and oral antibiotics lowered the risk of AL (OR 0.52, 0.30–0.92, *p* = 0.02), while MBP alone showed no statistically significant difference to no bowel preparation (OR 0.92, 0.63–1.36, *p* = 0.69). This study focused on left-sided colorectal resection and also demonstrated limited uptake of this strategy in contemporary international colorectal practice [37]. The study by Ambe et al. published in 2019 evaluated in patients undergoing elective CRC surgery the benefit of routine preoperative mechanical bowel preparation and oral non-absorbable antibiotics on reducing the risk of AL and obtained a rate of 4.0% compared with 9.1% in patients that received only MBP (*p* = 0.03) [40].

MBP is routinely employed to clear intestinal contents and reduce the overall bacterial burden in the gastrointestinal tract; however, it does not fully eradicate the microbiota, as mucosa-associated bacterial populations remain and may undergo compositional alterations. The surgical procedure induces an immediate and substantial shift in gut microbial composition, typically marked by a decrease in *Bifidobacterium* and *Lactobacillus* and an increase in *Escherichia coli* and *Staphylococcus*. These alterations are generally reversible within approximately 14 days, with the rate of recovery depending on the intensity of the preparation [27]. When MBP is administered in combination with oral antibiotics, its effects on the gut microbiota are more pronounced and sustained over time, influencing both short- and long-term microbial dynamics. In such cases, re-establishment of a baseline-like microbiota composition may require at least 30 days [41].

**Microbiota-targeted intervention—prebiotics/probiotics/synbiotics.** Considering the growing global burden of antibiotic resistance, the effects of antibiotic therapy (targeting not only pathogenic bacteria but also beneficial species, thereby disrupting gut microbiome homeostasis), and the influences of the microbiome dysbiosis and microbial metabolism on inflammation and gut barrier in the wound healing process after colorectal surgery, of significant interest is the possible use of a probiotic (in association or not with a prebiotic) to prevent postoperative complications, particularly AL [42]. Microbiota-targeted intervention agents act through complementary mechanisms aimed at restoring or modulating gut microbial balance and improving host intestinal function.

The International Society for Probiotics and Prebiotics defines probiotics as “live microorganisms that, when administered in adequate amounts, confer a health benefit on the host” [43,44]. Exact benefits depend on the strain and dose of the probiotic. They contribute to health by producing anti-inflammatory metabolites that reduce inflammatory signaling, inhibiting the growth of pathogenic microbes, supporting the repair of the intestinal barrier, and modulating the proliferation and differentiation of immune cells. In addition, probiotics can enhance the host’s synthesis of nutrients such as amino acids and vitamins. They also increase SCFA production and lower intestinal pH, creating an environment that discourages pathogen growth. Although probiotics generally do not permanently colonize the gut, sustained supplementation can alter the composition of the intestinal microbiota and contribute to its remodeling. Through ecological niche competition, including competition for nutrients, they may help alleviate disease symptoms [45].

Prebiotics, in contrast, are defined as substrates that are selectively utilized by microorganisms and confer health benefits. Examples such as inulin and GOS can be specifically fermented by gut microbes. By stimulating microbial metabolism, prebiotics help regulate both the gut microbiota and immune responses, having a broad and favorable safety profile. Compared with single- or multi-strain probiotic interventions, prebiotics may offer a more comprehensive approach to improving gut microbial balance [45].

Synbiotics are combinations of live microorganisms and substrates that are selectively utilized by host microbes to provide health benefits. They can be classified as either complementary—blending probiotics with prebiotics—or synergistic, where specific microbes are paired with substrates designed to be metabolized together to enhance their beneficial effects [45].

The authors of a 2022 study, designed as an umbrella meta-analysis to quantify the effect of probiotics or synbiotics use on preventing or reducing infectious complications after colorectal resections, observed that the eight existing meta-analyses before their study were of low methodological quality, with notable limitations in comprehensiveness and in reporting clinically relevant outcomes. The study continued as a systematic review and meta-analysis of 21 published randomized trials, encompassing 1961 patients, 973 in the intervention group (523 patients—synbiotics, 776 patients—probiotics) and 988 in the control group (whether placebo or standard care), questioning whether probiotics or synbiotics should be considered part of enhanced recovery programs. They revealed a significant reduction in overall infectious complications among patients receiving probiotics or synbiotics (12 trials; RR 0.59, 95% CI 0.47–0.75; *p* < 0.01), accompanied by low between-study heterogeneity (I^2^ = 15%). A statistically significant decrease in surgical site infections was also observed (11 trials; RR 0.70, 95% CI 0.52–0.95; *p* = 0.02), with no detected heterogeneity (I^2^ = 0%) in the intervention groups. The rate of AL was a secondary objective and no statistically significant differences were observed between groups—11 RCTs; RR 0.83, 95% CI 0.47–1.45; *p* = 0.53, with low to moderate heterogeneity across studies (I^2^ = 29%). They tried to address practical questions regarding the optimal formulation, type, and timing of probiotics or synbiotics in colorectal surgery. The optimal timing of administration could not be conclusively established, as both preoperative and perioperative regimens were shown to be effective in reducing overall infectious complications. Likewise, no definitive recommendation could be made regarding the most appropriate composition or number of probiotic strains, but most trials predominantly employed *Lactobacillus* and *Bifidobacterium*, occasionally in combination with *Streptococcus* or *Enterococcus*, species [8].

The most recent systematic review, published in April 2026 by Cashin and colleagues, examined the relationship between the gut microbiome and colorectal AL, highlighting distinct differences in microbial composition between patients who develop AL and those without postoperative complications. The analysis included 11 studies comprising 551 patients (143 with AL and 408 controls) and provided comprehensive data on microbiome changes across the perioperative period, including microbial diversity, taxonomic composition, and functional profiling. Alpha diversity, reflecting within-sample microbial richness, differed between AL and control groups in 7 of the 11 studies, with a consistent trend toward reduced diversity in AL samples; a more frequent significant difference was in alpha diversity between intra- and postoperative samples compared with preoperative samples (86% vs. 25%, respectively). *Lachnospiraceae* were found to be increased in patients with AL compared with controls in four studies. In contrast, *Streptococcus* spp. and *Citrobacter* spp. were identified as protective taxa, with a reduced incidence of AL observed in three studies. *Lactobacillus* spp. were also associated with a protective effect, being more frequently detected in control groups in two studies. Other bacterial taxa showed inconsistent associations with AL across studies, with variable increases or decreases in abundance, making their clinical relevance difficult to establish. Several specific bacterial taxa have been investigated as potential biomarkers or therapeutic targets, including *Alistipes onderdonkii*, *Parabacteroides goldsteinii*, and *Fusobacterium nucleatum* [46].

Phosphate modulated bacterial behavior through microbial phosphate-sensing pathways, and phosphate supplementation appeared to suppress this virulence response. In contrast, tranexamic acid exerted its effect by inhibiting the host fibrinolytic system, particularly plasminogen activation, a mechanism exploited by certain bacteria to enhance collagenase activity [47].

**Selection of microbiota intervention agents.** A probiotic strain is classified by its genus, species, and, where relevant, subspecies, along with a unique alphanumeric code that distinguishes it at the strain level. Recommendations for clinical use are based on human studies that correlate specific strains to the claimed benefits, describing levels of graded evidence. Following the general criteria, there are international practical guidelines for the clinical use of probiotics and prebiotics: The Clinical Guide to Probiotic Products developed by The World Gastroenterology Organisation (WGO) Global Guidelines [45]; there are Clinical Guides to Probiotic Products available in regions that meticulously review and publish annually to ensure constant information actualization (ex: Alliance for Education on Probiotics (AEProbio) in USA/Canada/UK—Applications, Dosage Forms and Clinical Evidence to Date—18th Edition, 2026 [48]; Recommendations of the Romanian Society of Gastroenterology and Hepatology (SRGH) for the Use of Probiotics and Prebiotics in Clinical Practice—2025 [49]). Each guideline offers recommendations for specific products containing single or multiple probiotic strains, as well as prebiotics, each with defined dosages and specific clinical indications. However, these clinical guidelines do not include any references to colorectal surgery, and particularly anastomotic leak.

The only mention of postoperative complications appears in the latest version of the World Gastroenterology Organisation guidelines (2023–2024), which include two product formulations with a level of evidence of 3, showing an effect in reducing the rate of postoperative septicemia and infectious complications. The recommended options are *Lactobacillus plantarum* CGMCC 1258, *L. acidophilus* 11, and *Bifidobacterium longum* 88, administered at a total daily dose of 2.6 × 10^14^ CFU, or a combination of *Lactobacillus acidophilus* NCFM, *L. rhamnosus* HN001, *L. paracasei* LPC-37, *Bifidobacterium lactis* HN019, along with fructooligosaccharides (6 g FOS plus 4 × 10^9^ CFU, twice daily).

These recommendations are based on three studies: A double-center and double-blind randomized clinical trial published by Liu et al. in 2015 [50] that administered probiotics for 6 days preoperatively and 10 days postoperatively for hepatic local resection in metastatic CRC patients and obtained a reduced concentration of serum zonulin as a marker of intestinal permeability and plasma endotoxin. The incidence of infectious complications such as septicemia in the probiotics group was lower, with an incidence of 59% vs. 88%, *p* = 0.008; hepatocytolysis syndrome also improved, and the overall gastrointestinal condition was better [51]. A meta-analysis published by Wang et al. in 2023 [52] also revealed improved intestinal mucosal barrier-related factors after microecological preparation administration: endotoxin (SMD, −2.6850 [−4.1399; −1.2301], *p* = 0.0003), diamine oxidase (SMD, −2.5916, [−3.4694; −1.7137], *p* < 0.0001) and plasma D-lactate (SMD, −5.4726, [−9.8901; −1.0551], *p*= 0.0152) [50].

The second source is a systematic review and meta-analysis of randomized controlled trials reported by Chowdhury et al. in 2020 [53], which evaluated the impact of perioperative treatment with probiotics or synbiotics on postoperative outcome in patients undergoing abdominal surgery. Across 34 randomized controlled trials including 2723 patients, 1354 received prebiotic or synbiotic preparations. Perioperative use of probiotics or synbiotics was associated with a significant reduction in infectious complications after abdominal surgery [RR = 0.56; 95%, CI = 0.46–0.69; *p* < 0.00001; *n* = 2723; I^2^ = 42%]. Synbiotics demonstrated a greater protective effect against postoperative infections compared with probiotics alone (synbiotics: RR 0.46; 95% CI 0.33–0.66; *p* < 0.0001; *n* = 1399; I^2^ = 53% vs. probiotics: RR 0.65; 95% CI 0.53–0.80; *p* < 0.0001; *n* = 1324; I^2^ = 18%). Although the analysis included a heterogeneous patient population, the large sample size provides strong evidence supporting the clinical benefits of probiotics, and particularly synbiotics [53].

The third study considered by WGO was developed by Flesch et al. and was also included in our analysis. As a safety consideration, the WGO recommends that individuals with compromised immune systems or significant underlying conditions limit probiotic use to strains and indications with well-established evidence of efficacy [45]. Selected examples of probiotic strains considered in surgical CRC patients and their action mechanisms are described in Table 2.

Considering the important role of the intestinal microbiome in anastomotic healing in patients with CRC undergoing surgery, as well as the intestinal dysbiosis induced by preoperative preparation and the possible use of antibiotics, the administration of a probiotic—potentially in combination with a prebiotic—may represent a strategy for preventing postoperative complications, particularly anastomotic leakage. Based on available prospective interventional studies and RCTs reported in the literature, their perioperative use represents a potential beneficial microbiota-targeted intervention in the prevention of postoperative complications, specifically AL. However, the specific strains, dosages, and methods of administration require careful evaluation.

## 3. Materials and Methods—Literature Review of RCT

In order to identify the potential best microbiota intervention agent (prebiotics/probiotics/synbiotics), we conducted an extensive literature review of RCTs that investigate the influence of a targeted microbiota intervention in the perioperative setting of radical surgical treatment of colorectal cancer patients.

The PICO framework of this review raised the following question: “In patients with colorectal cancer undergoing radical resection with anastomosis formation (P), does a microbiota-targeted intervention (I), compared with standard care or placebo (C), reduce the risk of postoperative complications, particularly anastomotic leak (O)?”. Our study population comprised colorectal cancer patients undergoing radical resection with anastomosis, the intervention investigated was microbiota-targeted interventions (prebiotics/probiotics/synbiotics) being controlled to placebo, no intervention, or standard care (usually MBP—mechanical bowel preparation ± OAB selective oral antibiotics). The outcome that we looked for was the incidence of anastomotic leak (AL) and other infectious postoperative complications. In order to obtain a high-certainty rating (considering the PICO framework) and further to formulate pertinent recommendations, we limited the study selection to RCTs. Effects on postoperative infectious complications and gastrointestinal motility were also noted.

The inclusion criteria are as follows: RCTs investigating targeted gut microbiome interventions with a specific (main or secondary) outcome documenting the rate of AL in CRC patients, but also colorectal surgery including CRC patients. Patients included were consenting adults that had undergone colorectal radical resection for primary tumors with anastomosis, regardless of risk factors and primary tumors’ specific locations (colon or rectum). We excluded from our research the studies that did not deliver any information regarding AL rates or that included patients with inflammatory bowel disease.

**Figure 1:**
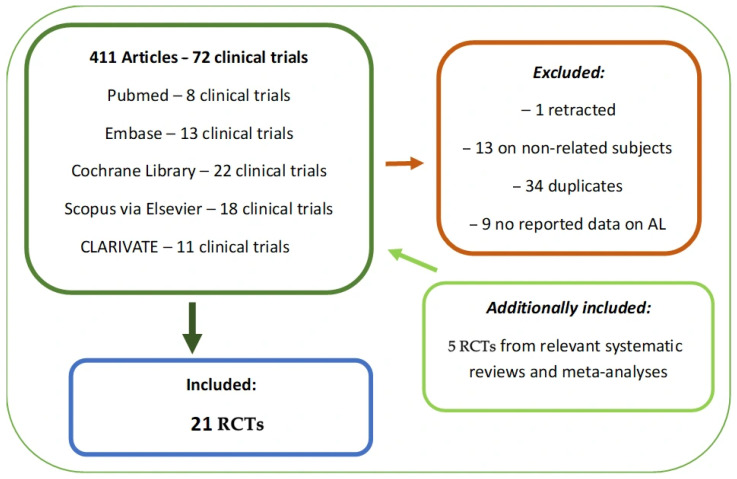
Search strategy and inclusion algorithm.

The study selection process was performed by two independent reviewers and disagreements between reviewers were resolved with the intervention of the project coordinator. We searched for clinical trials on the electronic databases PubMed, Embase, Scopus, and the Cochrane Central Register of Controlled Trials from inception to July 2025, with general search text (“colorectal cancer”[Title/Abstract] OR “colon cancer”[Title/Abstract] OR “rectal cancer”[Title/Abstract] OR “colorectal neoplasms”[MeSH]) AND (“surgery”[Title/Abstract] OR “surgical treatment”[Title/Abstract] OR “resection”[Title/Abstract] OR “colectomy”[MeSH] OR “proctectomy”[MeSH]) AND (“microbiome”[Title/Abstract] OR “gut microbiota”[Title/Abstract] OR “intestinal microbiota”[MeSH] OR “probiotics”[MeSH Terms] OR “prebiotics”[MeSH Terms] OR “paraprobiotics”[MeSH Terms] OR “postbiotics”[MeSH Terms] OR “synbiotics”[Title/Abstract] OR “fecal microbiota transplantation”[MeSH]) AND (“anastomotic leakage”[Title/Abstract] OR “anastomotic leak”[MeSH]). We identified 8 records in PubMed, 147 results with 13 clinical trials in Embase, 22 trials in the Cochrane Library, 227 results with 18 clinical trials records in Scopus, and 91 results with 11 clinical trials in CLARIVATE (Figure 1). In addition, RCTs cited in relevant systematic reviews and meta-analyses were manually screened and included if they met the predefined inclusion criteria but were not retrieved through the electronic search. After removal of duplicates and exclusion of non-randomized studies and trials lacking reported data on AL, relevant studies were reviewed, and key information was extracted using a structured data collection form. Extracted data included study characteristics as listed below. The extracted information was summarized and synthesized narratively to identify common themes, patterns, and areas of agreement or disagreement within the literature.

The following information was extracted:

- Study characteristics: author(s), year of publication, study design, and setting.
- Participant characteristics: sample size, age, tumor location, and eligibility criteria.
- Intervention details: type of microbiota-targeted intervention, dosage, duration, timing, and administration route.
- Comparator details: placebo, standard care, or alternative intervention.
- Outcomes: incidence of anastomotic leak, overall postoperative complications, surgical site infections, effects on gastrointestinal motility.
- Methodological quality: risk-of-bias information and relevant quality assessment data.
- Results: effect estimates, confidence intervals, *p*-values, and other outcome data required for synthesis.

Assessment of Study Heterogeneity among the included studies was performed qualitatively by examining differences in study design, patient populations, microbiota-targeted interventions, outcome definitions, and reported results. Variations in intervention type, treatment duration, perioperative protocols, and methods used to diagnose postoperative complications, particularly anastomotic leak, were considered when interpreting the findings. These sources of heterogeneity were taken into account during the narrative synthesis of the evidence.

Risk of bias was assessed according to The Cochrane Risk of Bias 2 (RoB 2) Tool.

## 4. Results

A total of 21 RCTs were included in the final analysis. Study characteristics consisting of location of study, sample sizes, male-to-female ratio are provided in Table 3. Specific dietary requirements are not generally noted: one study evaluated fiber intake based on pictures of the meal provided by patients. Dietary restrictions and administration of other probiotics/prebiotics or synbiotics used in trials were generally considered exclusion criteria. The results from the RCTs included are summarized in Table 4.

Four studies had a statistically significant reduction in incidence of AL. We also observed positive results in gastrointestinal motility and a reduction in infectious complications—surgical site infections, pulmonary infections and urinary tract infections.

In a randomized, double-blind, placebo controlled clinical trial published in 2017, using synbiotics in the particular nutritional and immunological status of patients with colorectal cancer undergoing elective radical resection, Flesch et al. observed a “competitive exclusion” as the acting mechanism in strains of *Lactobacillus rhamnosus*, *Bifidobacterium bifidum*, *Lactobacillus plantarum* and *Saccharomyces boulardii*. Postoperative infection rates were significantly reduced: among 91 patients, only one case of surgical wound infection occurred in the synbiotics group, compared with nine cases in the control group (*p* = 0.002), there were three cases of intra-abdominal abscess and four cases of pneumonia in the control group, whereas no such complications were observed in the synbiotics group (*p* = 0.001); and they suggested that perioperative oral administration of synbiotics may be a promising strategy for preventing surgical infections in patients with CRC [51].

When comparing the effects of administration of a single strain probiotic (*Bifidobacteria bifidum*) perioperatively with administration of antibiotics (kanamycin sulfate and metronidazole orally—3 doses) the day before surgery as a chemical bowel preparation, and a control group that received only standard mechanical bowel preparation in elective colon cancer surgical treatment, Sadahiro et al. observed in their randomized prospective trial that the most effective at preventing postoperative complications and particularly AL rates was using antibiotics in bowel preparation. They obtained a rate of incisional surgical site infection of 18.0% in the probiotics group—similar to 17.9% in the control group, and 6.1% in chemical bowel preparation—and a rate of AL of 12.0%, 7.4% and 1.0% respectively [66].

Liu et al. investigated the use of perioperative probiotics to preserve intestinal barrier function and reduce postoperative infectious complications after colorectal cancer surgery. Their findings showed that probiotics decreased intestinal permeability and bacterial translocation, likely by modulating serum zonulin levels and enhancing mucosal barrier integrity. Although surgical stress transiently impaired barrier function, patients receiving probiotics experienced faster recovery of intestinal permeability, which was associated with fewer postoperative infections and improved clinical outcomes [67,71].

Other effects of microbiome interventions may be helpful in adapting perioperative regimens, such as improved gastrointestinal motility and lower chance of hypoalbuminemia [67]. For anterior resection syndrome (ARS), probiotics have the capacity to improve flatus control, according to the results achieved in the RCT developed by Park et al. involving rectal cancer patients (31% in the probiotics group vs 10% in the placebo group *p*= 0.033) [52].

Considerable heterogeneity was anticipated due to differences in the strains of microbiota-targeted interventions, inclusion and exclusion criteria of each study, tumor location, use of MBP (±OAB) and definitions of anastomotic leak. Therefore, qualitative assessment findings were synthesized narratively, with emphasis on identifying consistent patterns across studies while acknowledging methodological and clinical variability.

Clinical heterogeneity was assessed by examining participant characteristics, study settings, interventions, comparators, and outcomes. Participant demographics were generally comparable across studies, with similar age and sex distributions (Table 3). Regarding tumor location, the included studies focused on colorectal cancer, with most enrolling both colon and rectal cancer patients, while a smaller number included only colon cancer cases. This may be explained by the frequent exclusion of patients receiving neoadjuvant therapy, which is the standard treatment for locally advanced low rectal cancer.

Considerable heterogeneity was observed in the study settings, as the studies were conducted across different countries and continents. However, the fact that many participating centers were affiliated with medical universities may support the overall quality and standardization of care provided.

The interventions, detailed in Table 4, varied substantially in their microbiota-targeted formulations. Most studies investigated multi-strain probiotic preparations, predominantly containing *Lactobacillus* and *Bifidobacterium* species, whereas only three studies evaluated single-strain probiotics. In addition, several studies incorporated prebiotics, including bioactive plant fibers, inulin, and fructooligosaccharides, while one study evaluated prebiotics alone. All microbiota-targeted interventions were administered orally using standardized protocols. The majority of studies employed perioperative administration; however, six studies administered the intervention exclusively preoperatively, and two studies administered it only postoperatively. The bacterial dose generally exceeded 10^8^ CFU per capsule.

Comparators consisted of either placebo or standard care, which commonly included MBP. In two studies, standard care also incorporated oral OAB. Four studies compared microbiota-targeted interventions not only with standard care but also directly between synbiotic/prebiotic and probiotic/antibiotic approaches.

Methodological heterogeneity was present across the included studies, primarily due to differences in sample size and follow-up duration. All included studies were randomized controlled trials; however, sample sizes ranged from 8 to 206 participants per study arm, representing a notable source of heterogeneity. Most intervention and control groups consisted of approximately 20–75 participants. Follow-up periods were generally similar, ranging from 30 days to 3 months postoperatively. Nevertheless, one study differed substantially by administering probiotics for one year and monitoring participants’ health status throughout the intervention period.

Outcome heterogeneity. Overall, the studies assessed broadly similar outcomes, including postoperative infectious complications, anastomotic leakage, and recovery of gastrointestinal function or motility. A subset of studies also investigated changes in gut microbiota composition. Despite this general consistency, outcome heterogeneity arose from variations in outcome definitions and reporting. Infectious complications were not uniformly defined across studies, and considerable variation existed in the classification of specific postoperative events. Although anastomotic leakage, surgical site infections, and intra-abdominal abscesses were reported in several studies, standardized definitions were not consistently applied. For example, some authors considered intra-abdominal abscesses to be a manifestation of anastomotic leakage resulting from the extravasation of intestinal contents into the peritoneal cavity, whereas others classified them as a distinct category of postoperative infectious complication. These differences in outcome definitions may have influenced the comparability of findings across studies.

Quality assessment is represented in Table 5, considering the PICO framework. Risk of bias was assessed according to The Cochrane Risk of Bias 2 presented in Table 6.

## 5. Discussion

The gut has been regarded as the “motor of multiorgan failure” due to its key involvement in initiating systemic inflammatory responses and postoperative sepsis. The human intestine contains a highly diverse microbial community consisting of approximately 500–1000 species of bacteria, yeasts, viruses, and parasites. The “gut microbiome” encompasses the collective genomes of these symbiotic microorganisms, which play vital roles in metabolic, nutritional, and biochemical processes essential for maintaining intestinal integrity and overall systemic homeostasis [74].

The human perioperative setting is inherently more heterogeneous, with factors such as comorbidities, nutritional status, and the use of systemic antibiotics potentially affecting the efficacy of microbiome-targeted therapies. These considerations emphasize the limitations of directly extrapolating preliminary preclinical findings to clinical practice and highlight the importance of careful, stepwise clinical evaluation [47].

US authors have suggested nutritional prehabilitation within Enhanced Recovery After Surgery (ERAS) programs, involving increased dietary fiber intake and reduced fat consumption. Experimental studies in mice have demonstrated that preoperative exposure to this dietary regimen can alter the gut microbiome and is associated with a reduced risk of anastomotic dehiscence. Available clinical evidence indicates that probiotics and synbiotics may be a promising adjunct for improving perioperative outcomes in colorectal surgery. The majority of RCT and meta-analyses report reduced infectious complications and enhanced recovery of intestinal function following their use. These findings are also supported by plausible biological mechanisms that underpin the clinical relevance of gut microbiota modulation [75].

The perioperative administration of a combination of probiotics and prebiotics for 7 days before surgery in patients undergoing treatment for CRC was shown to attenuate the inflammatory response, improve bowel function, reduce postoperative complications, decrease the cumulative duration of antibiotic therapy, and shorten hospital length of stay. Patients receiving probiotics demonstrated reductions in inflammatory markers, including the neutrophil-to-lymphocyte ratio (NLR), compared with placebo, along with lower levels of acute inflammation- and infection-related biomarkers. In addition, inflammatory cytokine profiles differed between groups: IFN-γ and IL-10, which possess anti-tumor and anti-inflammatory properties, increased in the probiotics group, whereas they decreased or remained unchanged in the placebo group [55].

Classical probiotic organisms are generally characterized by their capacity to produce beneficial metabolites such as SCF and lactic acid. However, the impact of probiotic-associated taxa on AL appears heterogeneous across studies. Overall, despite variability among taxa, probiotic organisms—particularly lactobacilli—were more frequently enriched in non-AL (protective) groups and reduced in AL cases. This observation is further supported by randomized controlled trials, which have linked probiotic supplementation with improved postoperative outcomes in colorectal cancer surgery.

In the comparative analysis published by Xu et al. in 2022 [76], the authors performed a network meta-analysis evaluating the efficacy of oral antibiotics, synbiotics, prebiotics, and probiotics in preventing postoperative infections following colorectal surgery. They included 10 RCTs involving 2531 participants. Four treatment strategies were compared. They concluded that oral antibiotic bowel preparation was the most effective strategy for preventing anastomotic AL, achieving the highest SUCRA score (0.927), with no significant global heterogeneity observed (I^2^ = 0%, τ^2^ = 0). In the subgroup analysis limited to laparoscopic surgery, oral antibiotics (compared with usual care, RR 0.67, 95% CI 0.28–1.64) and probiotics (compared with usual care, RR 0.50, 95% CI 0.11–2.31) appeared to be the most effective interventions. The authors also noted that the included trials used a wide range of probiotic and synbiotic formulations, which may have contributed to an underestimation of the treatment effects. Nevertheless, they considered it unlikely that this variability significantly affected the overall findings. Most studies employed preparations containing *Lactobacillus* and *Bifidobacteria*, the principal components of currently available probiotic-related products. In addition, the network meta-analysis evaluating infection rates demonstrated low heterogeneity (global I^2^ = 13.5%), suggesting only minimal between-study variability [76].

The probiotic combination of *Lactobacillus acidophilus* and *Bifidobacterium longum* has demonstrated several beneficial properties, including clinical safety, non-pathogenicity, genetic stability, resistance to gastrointestinal transit, antimicrobial activity, competitive inhibition of pathogenic microorganisms, and modulation of the intestinal immune response. In addition, these probiotic strains have been shown to support intestinal barrier integrity and contribute to the maintenance of gut microbial balance. Such characteristics make this probiotic combination a promising candidate for use in patients undergoing colorectal resection [77].

The effectiveness of treatment appears to be influenced by the bacterial genus, the quantity administered, and the number of viable probiotic organisms that reach and establish colonization within the colon. Patients receiving high-dose probiotics also demonstrated lower stool counts of *Enterobacteriaceae* and enterococci compared with the low-dose and placebo groups, although statistical significance was not reached for all comparisons. The proportion of patients with *Enterobacteriaceae* adherent to the colonic mucosa was significantly lower in the high-dose group (30%) than in the low-dose group (81.8%) and placebo group (70%) (*p* = 0.03 for high dose vs low dose). A similar trend was observed for enterococci adherence, although without statistical significance [77].

One of the most common exclusion criteria among the included randomized controlled trials was the use of antibiotic therapy during the preoperative period. It is important to distinguish this from perioperative antibiotic administration, which is considered standard clinical practice in colorectal surgery. Perioperative antibiotic therapy typically includes bowel decontamination protocols, often combined with MBP, using selective oral non-absorbable antibiotics, as well as intravenous prophylactic antibiotics administered around the time of surgery to reduce the risk of postoperative infectious complications. In contrast, preoperative antibiotic therapy administered for other indications may significantly alter the intestinal microbiota and therefore represent a potential confounding factor when evaluating the effects of microbiota-targeted interventions. Additionally, antibiotic therapy may be required postoperatively for the treatment of infectious complications, which could further influence microbiota composition and clinical outcomes.

In the prospective study published by Carlini et al. [78] in 2022 and 2023, a total number of 131 patients, including the first 60 cases experimentally treated, received the perioperative MIRACLe protocol and a historic retrospective group received standard ERAS preparation for surgery (only MBP and iv antibiotics the day of surgery). They tested the association of oral antibiotic prophylaxis, MBP and administration of probiotics (*Streptococcus thermophilus*, *Bifidobacterium breve*, *Bifidobacterium longum*, *Bifidobacterium infantis*, *Lactobacillus acidophilus*, *Lactobacillus plantarum*, *Lactobacillus paracasei* and *Lactobacillus delbrueckii* subsp. *Bulgaricus*). The probiotic was administered twice a day—five days before surgery and four days after surgery, and during surgery, with instillation of two doses directly at the site of anastomosis. The result was a decrease in AL incidence from 6.4% to 1.5% with a *p*-value of 0.028 (probiotics + MBP + OAB group 2/131 and control group 32/500). The results of their study provide proof of safeness of probiotic use in the perioperative setting of colorectal surgery, and show that the association of the three elements may decrease AL [78].

Unfortunately, the sample sizes within individual study groups were generally too small to allow meaningful subgroup analyses. Furthermore, in studies that included both colon and rectal cancer patients, outcomes were not reported separately for these subpopulations, precluding a more detailed analysis. Patients undergoing protective stoma formation were excluded from the included studies, as specified in the exclusion criteria of each trial.

Anastomotic technique was not included among the variables evaluated in this review. As discussed in the Introduction, numerous factors, including anastomotic technique, may influence the risk of anastomotic leakage. However, the objective of the present review was not to assess surgical risk factors for anastomotic dehiscence, but rather to investigate whether targeted microbiota interventions may contribute to the prevention of this complication despite the presence of established risk factors.

The studies included in our analysis, together with evidence from the literature, provide substantial support for the role of probiotics in modulating postoperative immune and inflammatory responses. They may play a beneficial role in preventing complications following colorectal surgery. Current evidence suggests that their use is associated with reduced infectious complications, modulation of the inflammatory response, and earlier recovery of intestinal function. Their administration appears to be safe and well tolerated. However, despite these promising findings, the optimal formulation and timing of administration have yet to be clearly established.

Our review limitations consist of considerable heterogeneity in intervention characteristics, which limits the comparability and interpretation of results across studies. Also the number of participants in the included studies was low and a larger group of risks represented exclusion criteria in some RCTs. Meaningful comparisons of clinical interventions should take into consideration the quality and consistency of perioperative care protocols. A proportion of the included studies did not provide detailed descriptions of their surgical care pathways, limiting our ability to evaluate the influence of contextual perioperative factors. Summarizing and reporting data on the effectiveness of an intervention alone, without consideration of implementation (e.g., prescription adherence) or effect modification related to patient factors (e.g., malnutrition, obesity), limits conclusions and generalizability [79]. Likewise, by pooling all patient groups, the beneficial and negative effects of the intervention can be missed. Also, few studies clearly defined AL or described the methods used for its assessment, and given the relatively low incidence of AL, this inconsistency may reduce confidence in the accuracy and comparability of the reported outcomes.

Future research should prioritize adherence to standardized consensus definitions of AL, include patients across the full spectrum of surgical risk, and ensure comprehensive reporting of clinically relevant outcomes. This narrative review was conducted to systematically evaluate microbiota-targeted interventions and identify the most promising therapeutic strategies for incorporation into a future prospective randomized clinical trial involving patients undergoing colorectal cancer resection. The primary objective of such a trial will be to determine whether an optimized microbiota-based intervention can reduce postoperative complications, particularly AL. This information will be instrumental in designing interventions that maximize efficacy while maintaining an acceptable safety profile.

## 6. Conclusions

Even though it is hard to make a clear recommendation based on existing studies, it is clear that perioperative administration is the most effective approach in restoring gut microbiota diversity, and a multi-strain agent containing *Lactobacillus* and *Bifidobacterium* could play a key role in preventing AL after surgical treatment in CRC patients. A prebiotic (inulin—GOS as a source of Butyrate, Phosphate) administered 5–14 days before surgery in one or two daily doses could be recommended as a preoperative preventive measure. MBP in association with selective OAB represents an effective preventive measure that is supported by the comprehensive literature review and RCT-supported data. Postoperative administration of probiotics may be beneficial in preventing AL, but it may also provide better immunological status and intestinal barrier function. Because of possible adverse effects and the effect on intestinal motility, postoperative day 2 could be a good start for oral administration of probiotics after surgery. Given the period of time needed to restore gut microbiota after MBP/antibiotic course and surgical intervention, probiotic administration should continue for 14 to 30 days.

In order to establish a valid recommendation for a targeted microbiota intervention as a preventive measure for AL in the particular case of CRC patients, further large-scale clinical studies are required. These studies should adhere to standardized consensus definitions of AL, include patients across all risk categories, and provide comprehensive documentation of clinical outcomes.

## Figures and Tables

**Table 1:** Intestinal microbiota composition and correlation with anastomotic healing and AL.

Microbiota Composition	Associated Mechanism	Effect on Anastomotic Healing/AL
*Enterococcus faecalis*	Selectively colonizes injured anastomotic tissue; produces collagenase; degrades type I collagen; activates MMP-9; binds and hyperactivates plasminogen (PLG); acquires increased virulence after antibiotic-induced dysbiosis	Major contributor to collagen degradation, impaired tissue repair, and increased risk of anastomotic leakage (AL)
*Pseudomonas aeruginosa*	Exhibits high collagenolytic activity; degrades type I collagen; increases epithelial invasion and tissue damage; adopts a more virulent phenotype after radiotherapy	Promotes extracellular matrix degradation and AL development
*Clostridium difficile*	Collagenase-producing pathogen that frequently overgrows after perioperative dysbiosis	Associated with increased AL risk and postoperative infectious complications
*Klebsiella pneumoniae*	Produces collagenases and proteases contributing to extracellular matrix degradation	Associated with increased incidence of AL
*Proteus mirabilis*	Produces collagen-degrading enzymes	Contributes to impaired anastomotic healing
*Bacteroides* spp.	Collagenase/protease production	Associated with excessive collagen degradation and AL
*Enterococcus* spp. (overall)	Local expansion at the healing anastomotic site following surgery; likely stimulated by collagen-rich environment	Frequently detected after AL diagnosis despite absence in intraoperative mucosal biopsies
*Bifidobacterium* spp. (high mucosal abundance)	May impair tissue perfusion and oxygenation around the anastomosis	Approximately fourfold increased risk of AL reported in one clinical study
*Alistipes onderdonkii*	Persists after bowel preparation and antibiotics; modulates mucosal inflammation	Associated with increased AL incidence in murine fecal microbiota transplantation (FMT) models
*Parabacteroides goldsteinii*	Modulates mucosal immune responses; survives bowel preparation and antibiotic exposure	Associated with reduced AL risk in murine models
Postoperative dysbiosis	Caused by fasting, bowel preparation, antibiotics, surgery, and postoperative physiological changes; favors overgrowth of pathogenic organisms (e.g., *E. faecalis*, *P. aeruginosa*, *C. difficile*)	Increases susceptibility to infection, delayed healing, and AL; restoration of microbiota may improve postoperative recovery
Balanced intestinal microbiota	Maintains epithelial barrier function, regulates immune homeostasis, limits pathogen overgrowth	Supports optimal postoperative healing and recovery
*Lactobacillus* spp.	Stimulates NOX1-dependent reactive oxygen species production, promoting epithelial proliferation and migration; inhibits pathogenic bacteria; maintains intestinal barrier integrity	Enhances anastomotic healing and protects against AL

**Table 2:** Examples of probiotic strains considered in surgical CRC patients and action mechanisms.

Probiotic Strain	Biological/Clinical Effect	Main Mechanism(s) of Action
*Lactobacillus rhamnosus* CNCM I-3690	Restores intestinal barrier integrity	Counteracts inflammation-induced intestinal permeability; regulates tight junction proteins (occludin and E-cadherin)
*Lactobacillus plantarum* BMCM12	Protects intestinal barrier	Secretes extracellular proteins that interfere with pathogen adhesion
*Lactobacillus plantarum*	Enhances epithelial barrier integrity	Increases occludin expression and promotes apical redistribution of occludin and ZO-1
*Lactobacillus plantarum* ZLP001	Preserves epithelial barrier function	Reverses enterotoxigenic Escherichia coli-induced reduction in claudin-1 and occludin
*Bifidobacterium polyfermenticus*	Exhibits anticancer activity	Produces bacteriocins that inhibit cancer cell growth
*Lactobacillus reuteri*	Protects against enteric pathogens and reinforces mucosal defense	Reduces enteropathogenic E. coli infection; inhibits Helicobacter pylori colonization; produces reuterin, which binds to the mucus layer and forms a protective barrier
*Akkermansia muciniphila* MucT	Reduces colitis-associated colorectal cancer and contributes to colorectal cancer prevention	Strengthens intestinal barrier; inhibits Th17-mediated IL-17 production

**Table 3:** An analysis of included RCTs studying effects of probiotics in CRC patients, but also colorectal surgery including CRC patients.

Author	CRC/Colorectal Surgery	Type of Intervention	Location	Number of Patients in Test Group vs. Control	Age of Patients in Test Group vs. Control	Sex of Patients in Test Group vs. Control (Male/Female)
1. Lee et al., 2023 [54]	Colon Cancer	Prebiotics	Chonnam National University Hwasun Hospital	79/82	60.7 ± 8.3/66.1 ± 11.1	Male 12 (75.0)/9 (56.3) Female 4 (25.0)/7 (43.8)
2. Park et al., 2020 [55]	Sigmoid Colon Cancer	Probiotics/ Prebiotics	Four medical centers in South Korea	31/29	61.03 (7.02)/60.10 (10.37)	Male 13 (41.94)/19 (65.52) Female 18 (58.06)/10 (34.48)
3. Bajramagic et al., 2019 [56]	CRC	Probiotics	Clinic of General and Abdominal Surgery of the UCCS	39/38	-	-
4. Kakaei et al., 2019 [57]	Colorectal Surgery	Probiotics	Sina and Imam Reza Hospitals of Tabriz University of Medical Sciences, Tabriz, Iran	50/50	50.08 ± 12.64/48.92 ± 11.23	Male Gender, *n* (%) 29 (58)/26 (52)
5. Polakowski et al., 2019 [58]	CRC	Synbiotics	Erasto Gaertner Cancer Hospitalin Curitiba, Brazil	36/37	60.9 (±6.7)/58.9 (±6.3)	Sex, male (%) 20 (55)/19 (51)
6. Flesh et al., 2017 [51]	CRC	Synbiotics	Coloproctology Service of the Porto Alegre Clinics Hospital	49/42	64.5 (±11.4)/61.1 (±13.4)	Female 31 (54.8%)/23 (63.3%) Male 18 (45.2%)/19 (36.7%)
7. Komatsu et al., 2016 [59]	Colorectal Surgery	Synbiotics	Nagoya Daini Red Cross Hospital	173/206	66.7 (±11.6)/67.7 (±10.7)	Male 92 (54.8%)/118 (60.8%) Female 76 (45.2%)/76 (39.2%)
8. Krebs et al., 2016 [60]	CRC	Synbiotics/Prebiotics	University Hospital Maribor, Slovenia	Synbiotics 20/Prebiotics 18/MBP 16	Synbiotics 62 (43–87)/ Prebiotics 64 (46–81)/ MBP 67 (52–78)	Male/female Synbiotics 11/7 Prebiotics 13/7 MBP 9/7
9. Mizuta et al., 2016 [61]	Colorectal Surgery	Probiotics	Mitoyo General Hospital Takamatsu, Japan	29/31	71.2 ± 9.5/68.9 ± 10.4	Male/female 15/14 vs. 20/11
10. Tan et al., 2016 [62]	CRC	Probiotics	University of Malaya Medical Centre	20/20	64.3 ± 14.5/68.4 ± 11.9	Male 11/13 Female 9/7
11. Yang et al., 2016 [63]	CRC	Probiotics	Shanghai Jiao Tong University Affiliated Sixth People’s Hospital	30/30	62.17 ± 11.06/63.90 ± 12.25	Female 18/15 Male 12/15
12. Aisu et al., 2015 [64]	CRC	Probiotics	Fukuoka University Hospital	75/81	68.0 ± 13.8/69.1 ± 11.3	M/F: 44/37 vs. 47/28
13. Kotzampassi et al., 2015 [65]	Colorectal Surgery	Synbiotics	AHEPA University Hospital of Thessaloniki	84/80	65.9 ± 11.5/66.4 ± 11.9	Male/female 57 (67.5)/27 (32.1) vs. 58 (72.5)/22 (27.5) POSSUM score
14. Sadahiro et al., 2014 [66]	Colon Cancer	Probiotics	Tokai University	Probiotics 100/Placebo 95/Antibiotics 100	Probiotics 67 ± 9/Placebo 67 ± 11/Antibiotics 66 ± 12	Male/female Probiotics 49/51/Placebo 56/43/Antibiotics 51/44
15. Liu et al., 2013 [67]	CRC	Probiotic	Shanghai Sixth People’s Hospital, affiliated with Shanghai JiaoTong University in Shanghai, and the Sixth Affiliated Hospital of Sun Yat-sen University in Guangzhou	75/75	66.06 6 ±1 1.02/62.28 6 ± 12.412	Male 38/40 Female 37/35
16. Pellino et al., 2013 [68]	CRC	Probiotics	Second University of Naples, Italy	10/8	71.5 (±2.1)/72.9 (±1.6)	(M/F) 5/5 vs. 4/4
17. Mangell et al., 2012 [69]	Colon resection	Probiotics	Lund University	32/32	74 (70–80)/70 (64–79)	Male/female 16/16 vs. 20/12
18. Zhang et al., 2012 [70]	CRC	Probiotics	Xin-Hua Hospital, Shanghai Jiaotong University	30/30	67.5 (45.0–87.0)/61.5 (46.0–82.0)	Male/female 10/20 vs. 14/16
19. Liu et al., 2011 [71]	CRC	Probiotics	Shanghai Sixth People’s Hospital, Shanghai Jiao Tong University	50/50	65.3 ±11.0/65.7 ±9.9	Male/female 31/19 vs. 28/22
20. Horvat et al., 2010 [72]	Colon cancer	Synbiotics/Prebiotics	University Clinical Center Maribor, Maribor, Slovenia	Synbiotics 20/Prebiotics 28/Control 20	Synbiotic 62 (42–86)/Prebiotics 62 (29–80)/Control 65 (52–78)	Male/female Synbiotic 9:11/Prebiotics 10:18/Control 11:9
21. Reddy et al., 2007 [73]	Colon surgery	MBP/MBP + OAB/MBP + OAB + Synbiotics/ OAB + Synbiotics	Scarborough Hospital	MBP 24/MBP + OAB 22/MBP + OAB+ Synbiotics 20/ OAB + Synbiotics 22	MBP 68.5 (61–75)/MBP + OAB 72.5 (53–81)/MBP + OAB+ Synbiotics 68.5 (62.5–74)/ OAB+ Synbiotics 65 (56–75)	Male/female MBP 11:13/MBP + OAB 13:9/MBP + OAB+ Synbiotics 9:11/ OAB+ Synbiotics 11:11

**Table 4:** RCTs studying effects of probiotics in CRC patients, but also colorectal surgery including CRC patients, with results on influence on AL rates as our main focus, and on gastrointestinal motility and infectious complications as a secondary interest.

Author	CRC/Colorectal Surgery	Type of Intervention	Formulation	Timing (Duration)	Dosage (CFU/g)	MBP ± Oral Ab	Effect on Anastomotic Leak	Number of Events (Intervention Group/Control Group)	Effect on Gastrointestinal Motility	Effect on Infectious Complications
1. Lee et al., 2023 [54]	Colon Cancer	Prebiotics	Arginine and omega-3 fatty acids	Pre (7 d)	400 mL/day	NA	No significant difference	0 leaks in study population	NA	No significant differences
2. Park et al., 2020 [55]	Sigmoid Colon Cancer	Probiotics/ Prebiotics	*Bifidobacterium animalis* subsp. lactis HY8002, *Lactobacillus casei* HY2782 *Lactobacillus plantarum* HY7712 vs. 350 mg of xylooligosaccharides and 36 mg of fructooligosaccharides	Post (4 weeks)	(10^8^ CFU), (5 × 10^7^ CFU), (5 × 10^7^ CFU) respectively and 386 mg prebiotic	MBP	No significant difference	AL: Probiotics: 0/29 Placebo: 1/31 Postoperative complications: probiotics: 2 (6.0%) vs placebo: 10 (28.5%) *p* = 0.024	Flatus control significantly improved in major Anterior Resection Syndrome	Decreased, with no significant differences 2/29 vs. 5/31
3. Bajramagic et al., 2019 [56]	CRC	Probiotics	(*Lactobacillus acidophilus*, *Lactobacillus casei*, *Lactobacillus plantarum*, *Lactobacillus rhamnosus*, *Bifidobacterium lactis*, *Bifidobacterium bifidum*, *Bifidobacterium breve*, *Streptococcus thermophilus*)	Post (1 year from POD 3)	2 capsules daily (2 × 1) for 30 days, then 1 capsule daily (1 × 1) for two weeks each subsequent month, for a total duration of one year	NA	No significant difference	Anastomosis loosening—probiotics: 2/39 (5.1%) vs. control: 5/78 (12.8%) *p*=0.431 Intraabdominal abscess: probiotics: 5 (12.8%) vs control: 7 (17.9%) *p*=0.530	Reduced postoperative ileus	No effect
4. Kakaei et al., 2019 [57]	Colorectal Surgery	Probiotics	*Lactobacillus casei* and *Lactobacillus acidophilus*, *Lactobacillus plantarum*, *Bifidobacterium breve*, *Bifidobacterium longum*, and *Streptococcus thermophilus*	Pre (7 d) and post (30 d)	(1.75 × 10^10^ CFU), (0.5 × 10^10^ CFU), (1.75 × 10^10^ CFU), (1.75 × 10^10^ CFU), (1.5 × 10^10^ CFU) respectively	MBP + OAB	No significant difference	Probiotics: 0/50 vs. control: 0/50 *p* = 1	NA	No significant difference (probiotics: 3 (6%) vs. placebo: 5 patients (10%) *p* = 0.46)
5. Polakowski et al., 2019 [58]	CRC	Synbiotics	Fructooligosaccharide, *Lactobacillus acidophilus* NCFM, *L. rhamnosus* HN001, *L. casei* LPC-37, and *Bifidobacterium lactis* HN019	Pre (7 d)	2.6 × 10^14^ CFU/2 g	MBP	Reduced	Synbiotics: 0/36 (0%) vs. control: 4/37 (10.8%)	Postoperative movement was earlier	Decreased (synbiotics: 1 (2.8) vs. placebo 7 (18.9) *p*= 0.02)
6. Flesh et al., 2017 [51]	CRC	Synbiotics	*Lactobacillus acidophilus*, *Lactobacillus rhamnosus*, *Lactobacillus casei*, *Bifi dobacterium* And fructooligosaccharide (FOS) 6 g	Pre (5 d) and Post (14 d)	3 × 10^9^ + 6 g prebiotic	MBP	Reduced	Synbiotics: 0/49 vs. control 3/42	No effect	Decreased —synbiotics: 1/49 vs. control 9/42 *p* = 0.002
7. Komatsu et al., 2016 [59]	Colorectal Surgery	Synbiotics	(Galactooligosaccharides, *Lactobacillus casei*, and *Bifidobacterium breve*)	Pre (7–11 d) and post (2–7 d)	5 × 10^10^ CFU/180 mL	MBP	No significant difference	Synbiotics: 12/173 vs. control: 12/206	NA	No effect
8. Krebs et al., 2016 [60]	CRC	Synbiotics/ Prebiotics	*Pediacoccus pentosaceus* 5–33:3, *Leuconostoc mesenteroides* 32–77:1, *Lactobacillus paracasei* subsp. paracasei 19, and *Lactobacillus plantarum* 2362. + betaglucan, inulin, pectin and resistant starch	Pre (3 d)	10^11^ of each + 2.5 g of each of the four fermentable fibers	MBP—control group	No significant difference	Synbiotics: 0/20 vs. prebiotics 0/18 vs. MBP 0/16	No significant difference	No significant difference
9. Mizuta et al., 2016 [61]	Colorectal Surgery	Probiotics	(*Bifidobacterium longum* BB536)	Pre (7–14 d) and post (14 d)	5 × 10^10^ CFU/2 g	MBP	No significant difference	Probiotics: 31/control: 31, 9.7% vs. 17.2%, *p* > 0.05	NA	No effect
10. Tan et al., 2016 [62]	CRC	Probiotics	*Lactobacillus acidophilus* (BCMCTM12130), *Lactobacillus casei* (BCMCTM12313), *Lactobacillus lactis* (BCMCTM12451), *Bifidobacterium bifidum* (BCMCTM02290), *Bifidobacterium longum* (BCMCTM02120), *Bifidobacterium infantis* (BCMCTM02129)	Pre (7 d)	30 billion colony-forming units	NA	No significant difference	Probiotics 4/20 vs. placebo 8/20, *p* = 0.3		No effect
11. Yang et al., 2016 [63]	CRC	Probiotics	*Bifidobacterium longum*, *Lactobacillus acidophilus*, and *Enterococcus faecalis*	Pre (5 d) and post (7 d)	1.0 × 10^7^ CFU/g—2 gX3/zi	MBP	No significant difference	Probiotics: 30/placebo: 30—3.3–6.7%, *p* = 1.00	Improved, lowered incidence of diarrhea	Lowered bacteriemia
12. Aisu et al., 2015 [64]	CRC	Probiotics	*Enterococcus faecalis* T110, *Clostridium butyricum* TO-A and *Bacillus mesentericus* TO-A	Pre (3–15 d) and post (? d)	2 mg + 10 mg + 10 mg	MBP	Reduced	75/81, 2.7% vs. 4.9%, *p* = 0.016	NA	Reduced—6.7% vs. 19.8% (*p* = 0.016).
13. Kotzampassi et al., 2015 [65]	Colorectal surgery	Synbiotics	(*Lactobacillus acidophilus*, *Lactobacillus plantarum*, *Bifidobacterium lactis* and *Saccharomyces boulardii*)	Pre (1 d) and Post (15 d)	(1.75 × 10^9^ CFU), (0.5 × 10^9^ CFU), (1.75 × 10^9^ CFU) and (1.5 × 10^9^ CFU), respectively—4 Capsules	MBP	Reduced	Probiotics: 1/84 vs. control: 7/80 (1.2% vs. 8.8%) *p* = 0.031 OR = 0.13 (CI = 0.01–0.99)	Reduced first flatus and defecation time	Decreased surgical site infection and pneumonia infection
14. Sadahiro et al., 2014 [66]	Colon Cancer	Probiotics	(*Bifidobacterium bifidum*)	Pre (7 d) and post (10 d)	10 billion × 3/day	MBP	No significant difference	Probiotics 100/placebo 95—7.4% vs. 12%, *p* = 0.56, probiotics 7 (7.4%) vs. antibiotics 1/100 (1.0%) vs. control 12/100 (12.0%) *p* = 0.004	NA	No effect
15. Liu et al., 2013 [67]	CRC	Probiotics	*Lactobacillus plantarum* 1258, *Lactobacillus acidophilus*-11 and *Bifidobacterium longum*-88	Pre (6 d) and post (10 d)	(10^11^ CFU), (7 × 10^10^ CFU), (5 × 10^10^ CFU) respectively	MBP	No significant difference		Improved peristalsis, incidence of diarrhea	Lowered intestinal permeability, lower chance of hypoalbuminemia
16. Pellino et al., 2013 [68]	CRC	Probiotics	*Streptococcus thermophilus* DSM 24731, *Bifidobacteria* (*B. longum* DSM 24736, *B. breve* DSM 24732, *B. infantis* DSM 24737), *Lactobacilli* (*L. acidophilus* DSM 24735, *L. plantarum* DSM 24730, *L. paracasei* DSM 24733, *L. delbrueckii* subsp. *Bulgaricus* DSM 24734)	1 day after discontinuation of antibiotics to 4 weeks—perioperative antibiotic therapy 24 h iv	900 × 10^9^ CFU × 2/day	NA	No significant difference	Complications Probiotics 1/10 vs. Placebo 1/8	Lower bowel frequency	NA
17. Mangell et al., 2012 [69]	Colon resection	Probiotics	(*Lactobacillus plantarum* 299 v)	Pre (8 d) and post (5 d)	10^11^ CFU	MBP	No significant difference	Probiotics: 32 vs. Placebo: 1/32 (intrabdominal abscess)	No effect	No effect
18. Zhang et al., 2012 [70]	CRC	Probiotics	(*Bifidobacterium longum*, *Lactobacillus acidophilus*, and *Enterococcus faecalis*)	Pre (3 d)	10 ^8^ CFU/g × 3/day	MBP + OAB	No significant difference	Probiotics 30/Placebo 30—0% vs. 3.3%, *p* = 0.492	Better recovery of bowel function	Decreased bacteremia and septicemia
19. Liu et al., 2011 [71]	CRC	Probiotics	*Lactobacillus plantarum* CGMCC 1258, *Lactobacillus acidophilus* LA-11, *Bifido-bacterium longum* BL-88	Pre (6 d) and post (10 d)	2.6 × 1014 cfu/2 g	MBP	No significant difference	Probiotics: 0/50 vs. Placebo: 0/50	Improved peristalsis, incidence of diarrhea	Decreased bacteremia and septicemia
20. Horvat et al., 2010 [72]	Colon cancer	Probiotics	(*Lactobacillus Pediacoccus pentosaceus* 5–33:3, *Lactobacillus Leuconostoc mesenteroides* 32–77:1, *Lactobacillus paracasei* subsp. paracasei 19, *Lactobacillus plantarum* 2362)/prebiotics (bioactive plant fibers: betaglucan, inulin, pectin, resistant starch)	Pre (3 d)	40 billion lactobacilli plus 10 g of bioactive plant fibers	MBP—Control group	No significant difference	Synbiotics: 0/20 vs. Prebiotics: 0/28 vs. MBP: 0/20	Reduced first flatus and defecation time	No effect
21. Reddy et al., 2007 [73]	Colon surgery	MBP/MBP + OAB/MBP + OAB+ Synbiotics/ OAB + Synbiotics	*Lactobacillus acidophilus* La5, *Lactobacillus bulgaricus*, *Bifidobacterium lactis* BB-12 and *Streptococcus thermophilus*	NA	4 × 10^9^ CFU × 3/day, 15 g oligofructose powder twice daily		No significant difference	MBP (2/24)/MBP + OAB (0/22)/MBP + OAB + Synbiotics (1/20)/OAB + Synbiotics (0/22) *p* = 0.315	No significant difference	No significant difference

**Table 5:** Qualitative assessment of included RCTs using the PICO framework.

Author	Year	Methodological Description of Study Design	Population Clearly Stated	Intervention Stated	Control Group	Outcomes	Fecal Microbiota Analysis	Colon	Rectum	Surgical Outcomes	Study Limitations Stated
Lee [54]	2023	yes	yes	yes	yes	yes	yes	yes	no	no	no
Park [55]	2020	yes	yes	yes	yes	yes	yes	yes	no	yes	yes
Polakowski [58]	2019	yes	no	yes	yes	yes	no	yes	yes	no	no
Kakaei [57]	2019	yes	no	yes	yes	yes	no	yes	yes	yes	yes
Bajramagic [56]	2019	yes	yes	yes	yes	yes	no	yes	yes	yes	no
Flesh [51]	2017	yes	no	yes	yes	yes	no	yes	yes	yes	no
Yang [63]	2016	yes	yes	yes	yes	yes	no	yes	yes	yes	yes
Tan [62]	2016	yes	yes	yes	yes	yes	no	yes	yes	yes	no
Mizuta [61]	2016	yes	yes	yes	yes	yes	yes	yes	yes	yes	yes
Krebs [60]	2016	yes	yes	yes	yes	yes	yes	yes	yes	yes	no
Komatsu [59]	2016	yes	yes	yes	yes	yes	yes	yes	yes	yes	no
Kotzampass [65]	2015	yes	yes	yes	yes	yes	no	yes	yes	yes	no
Aisu [64]	2015	yes/no randomization	yes	yes	yes	yes	no	yes	yes	yes	no
Sadahiro [66]	2014	yes	yes	yes	yes	yes	no	yes	no	yes	no
Pellino [68]	2013	yes	yes	yes	yes	yes	no	yes	no	yes	no
Liu [67]	2013	yes	yes	yes	yes	yes	yes	yes	no	yes	yes
Zhang [70]	2012	yes	yes	yes	yes	yes	yes	yes	yes	yes	no
Mangell [69]	2012	yes	yes	yes	yes	yes	yes	yes	no	yes	yes
Horvat [72]	2010	yes	yes	yes	yes	yes	no	yes	no	no	no
Liu [71]	2011	yes	yes	yes	yes	yes	yes	yes	yes	yes	yes
Reddy [73]	2007	yes	no	yes	yes	yes	yes	yes	yes	no	no

**Table 6:** Risk of bias of included RCTs according to Cochrane Risk of Bias 2 (RoB 2) Tool—x High, - Some concerns, + Low.

Author	Bias Arising from the Randomization Process	Bias Due to Deviations from Intended Interventions	Bias Due to Missing Outcome Data	Bias in Outcome Measurement	Bias in Selection of the Reported Result	Overall
1. Lee et al., 2023 [54]	+	-	x	+	-	x
2. Park et al., 2020 [55]	+	+	+	+	+	+
3. Bajramagic et al., 2019 [56]	+	+	x	-	+	x
4. Kakaei et al., 2019 [57]	+	+	+	+	+	+
5. Polakowski et al., 2019 [58]	+	+	+	+	+	+
6. Flesh et al., 2017 [51]	+	+	+	+	+	+
7. Komatsu et al., 2016 [59]	+	+	+	+	+	+
8. Krebs et al., 2016 [60]	-	+	+	+	+	-
9. Mizuta et al., 2016 [61]	+	+	+	-	+	-
10. Tan et al., 2016 [62]	+	+	+	+	-	-
11. Yang et al., 2016 [63]	+	+	+	+	+	+
12. Aisu et al., 2015 [64]	x	+	+	+	+	x
13. Kotzampassi et al., 2015 [65]	+	+	+	+	+	+
14. Sadahiro et al., 2014 [66]	+	+	+	+	+	+
15. Liu et al., 2013 [67]	+	+	+	+	-	-
16. Pellino et al., 2013 [68]	+	+	+	-	+	-
17. Mangell et al., 2012 [69]	+	+	+	+	+	+
18. Zhang et al., 2012 [70]	+	+	-	+	+	+
19. Liu et al., 2011 [71]	+	+	+	+	+	+
20. Horvat et al., 2010 [72]	+	+	+	-	+	-
21. Reddy et al., 2007 [73]	+	+	+	+	+	+

## Data Availability

No new data were created or analyzed in this study. Data sharing is not applicable to this article.

## Abbreviations

The following abbreviations are used in this manuscript:

AL	Anastomotic leakage
CRC	Colorectal cancer
RCTs	Randomized controlled trials
MMP-9	Matrix metalloproteinase-9
CoReAL	The International Consensus on Reporting Anastomotic Leaks After Colorectal Cancer Surgery
ISREC	International Study Group of Rectal Cancer
CRP	C-reactive protein
PCT	Procalcitonin
SCFAs	Short-chain fatty acids
GOS	Galacto-oligosaccharides
HDAC	Histone deacetylase
PLG	Human plasminogen
FMT	Fecal microbiota transplantation
MBP	Mechanical bowel preparation
OAB	Selective Oral Antibiotics
NOX1	NADPH oxidase 1
PEG	Polyethylene glycol
ESCP	European Society of Coloproctology
WGO	The World Gastroenterology Organisation
AEProbio	Alliance for Education on Probiotics
RR	Relative risk
CI	Confidence interval
ARS	Anterior resection syndrome
ERAS	Enhanced Recovery After Surgery
NLR	Neutrophil-to-lymphocyte ratio
